# The global diet: trade and novel infections

**DOI:** 10.1186/1744-8603-1-4

**Published:** 2005-04-22

**Authors:** Jill R Hodges, Ann Marie Kimball

**Affiliations:** 1 Health Services, University of Washington School of Public Health and Community Medicine, Seattle, USA

## Abstract

Practices designed to meet the demands of global trade can amplify food safety problems. Ever-increasing pressure to churn out more product and better sides of beef has generated processes that compromise existing safety measures. Among the concerns are intensified food production, use of antimicrobials and hormones as growth promoters, and poor sanitary infrastructure in some food producing countries. Accompanying the innovations designed to serve the diversifying global palate are emerging and re-emerging infectious diseases, or "trade-related infections." The joint efforts of international public health and industry are required to effectively address these growing health challenges.

## Review

As food production and distribution practices evolve to keep pace with rapidly diversifying consumer demand and international competition, new pathogens are emerging and long-known microbes are expanding their reach. Resilient bacteria such as *Salmonella*, *Escherichia coli*, *Listeria monocytogenes *and *Cyclospora cayetanensis *insinuate themselves into fruit, vegetables, poultry, beef and dairy products as they circulate around the globe, generating "trade-related infections" [1] (see Table 1). The pathogens may survive or multiply in foodstuffs and spread to humans and other vertebrates along the way. As soon as food safety measures address one problematic infection pathway, the microbes appear somewhere they have never been detected before. Food and waterborne diseases account for an estimated 2.1 million deaths annually in developing countries, and foodborne disease affects up to 30 percent of the population in industrialized countries [2]. Food safety is a "farm to fork" effort [3], and in the modern world of transnational integrated food supply and global trade, the distance between those two points has increased. International commerce has tripled over the last two decades [4]. In 2002, agricultural products accounted for about nine percent of international trade dollars – worth some $583 billion [5]. As the global exchange of agricultural products and the accompanying health risks proliferate, international efforts to control trans-border disease transmission are becoming increasingly important. This article will explore emergent and re-emergent foodborne infections that are coincident with the rise in global agricultural trade. Although the recent Bovine Spongiform Encephalopathy (BSE) and variant Creutzfeldt-Jakob Disease (vCJD) epidemics are compelling examples of this dynamic, this review focuses on enteric diseases and will not include prion disease.

**Table 1:** The nexus of global trade and foodborne pathogens.

**Pathogen**	**Origin**	**Trade-related interaction**
*Salmonella*	Described in the late 1880s in swine. Subsequently recognized in humans, poultry, cattle, rodents and exotic pets.	Use of antimicrobials in livestock in response to heightened global competition has contributed to emergence of antimicrobial-resistant strains such as *S. typhimurium *DT104 and *S. *Newport-MDRAmpC.
*Escherichia coli *O157:H7	Identified as a pathogenic agent in humans in 1982. Hosts include cows, deer, sheep, horses, pigs and dogs.	Intensified production and far-reaching distribution channels in the meat industry enable widespread dissemination in vehicles such as ground beef.
*Cyclospora cayetanensis*	First documented cases observed in humans 1977. Only known host is humans.	Hardy oocysts are transported on produce exported to geographic regions where the parasite previously had been largely unknown.
*Listeria monocytogenes*	Detected in 1926 in rabbits and guinea pigs, identified as a source of human infection in 1929 and perinatal contamination in 1936.	Increased popularity on the global market of raw milk cheeses and ready-to-eat products contributed to surge in listeriosis.

### Salmonella: New Pathways and Strains

While *Salmonella *is among the longest-known and most common foodborne pathogens, the Salmonellosis outbreaks stemming from the growing global exchange have revealed areas in which current knowledge is limited or outdated. *Salmonella*, traditionally linked primarily with poultry, eggs, raw meat, and dairy products, recently has been associated with a growing number of nuts, vegetables and fruits. Meanwhile, *Salmonella *has demonstrated an unanticipated hardiness and increasingly is emerging in antimicrobial-resistant strains.

*Salmonella *is found worldwide, with different serotypes prevalent in different regions – making it possible to track the incursion of new strains that may be linked to international commerce. The pathogen readily reproduces in a variety of foods, especially milk, quickly reaching a high infectious dose if the food is not refrigerated. In the right conditions, *Salmonella *can persist in the environment for weeks, even months. Infection commonly results from inadequately processed or undercooked eggs, poultry, dairy or meat. But humans also can transmit the bacterium through fecal-oral contact, and fruits and vegetables can be infected by contaminated water, work surfaces and utensils. *Salmonella *has caused massive outbreaks of illness. In 1994, an estimated 224,000 people across the U.S. developed *Salmonella enteritidis *infections after consuming ice cream apparently contaminated during transport in tankers that previously had carried nonpasteurized eggs [6]. The cross contamination highlights the tenacity of *Salmonella *and the low doses required for infection.

#### New Pathways

The mechanisms of contamination of newly identified sources of *Salmonella *infection are in some cases surprising, and in others, poorly understood. Among the previously unsuspected vehicles are seeds destined to become sprouts. During one of the many steps in their production, from growth to harvest to shipment, the seeds can be contaminated, primarily by contact with animal feces. The dry conditions under which the seeds are stored enable *Salmonella *and other pathogens to survive for months. All the while, the seeds sheltering the bacteria appear unharmed. During sprouts' warm, moist germination process, the microbes thrive. Sprouts have been linked to a series of outbreaks across North America, Western Europe and Japan [7]. A 1995 outbreak of *Salmonella *Stanley, for instance, spanned 17 U.S. states and Finland. The infection ultimately was traced to a distributor in the Netherlands who had obtained alfalfa seeds from Italy, Hungary and Pakistan; investigators were not able to determine which source was implicated in these outbreaks [8].

The contamination pathway in the first known case of an outbreak associated with imported mangoes is particularly paradoxical. In 1999, at least 78 people in 13 U.S. states became ill from a common strain of *Salmonella enterica*; 15 patients were hospitalized and two died [9]. Investigators traced the mangoes back to a farm in Brazil. They discovered that surprisingly, no Europeans who had consumed mangoes from the same farm had fallen ill. Investigators deduced that the mangoes destined for the U.S. likely had absorbed the microbe during a hot water treatment to repel fruit flies. The treatment was required to meet U.S. standards barring produce carrying the Mediterranean fruit fly – standards the Europeans did not impose.

#### Antimicrobial resistance

While overall rates of *Salmonella *have been dropping in the U.S. since 1996, the rates of drug-resistant strains have been on the rise. More than a quarter of *Salmonella *isolates are resistant to at least one antimicrobial; a significant portion has multiple resistances[10]. Infections resulting from these strains are not only difficult to treat because of their resistance to drugs, they also can cause more serious illnesses and hospitalizations [11,12].

Among the most prominent drug-resistant strains is *Salmonella typhimurium *definitive phage type 104 (*S. typhimurium *DT104), which began appearing with increasing frequency in the 1990s after fluoroquinolones were approved for use in food-producing animals. *S. typhimurium *DT104 is unresponsive to a handful of common antimicrobials, including ampicillin, chloramphenicol, streptomycin, sulfonamides and tetracyclines[13]. First detected in the United Kingdom in 1984, it quickly became one of the most commonly reported strains of *Salmonella *in England and Wales, linked with consumption of chicken, pork sausage, meat cakes, and eventually, beef [14]. While isolated cases DT104 appeared in the U.S. in the early 1980s, by the mid-1990s, the pathogen had become widespread [15]. More recently, a drug-resistant strain of *Salmonella *Newport, Newport-MDRAmpC, has emerged in the U.S. Newport-MDRAmpC is resistant or less susceptible to at least nine antimicrobials, including those on DT104's list and cephalosporins, which often are used to treat children with serious cases of salmonellosis[16].

Although most drug resistant infections in people have resulted from the use of antibiotics in human medicine, another contributing factor is antimicrobial applications in food producing animals. Once the bacteria in the animals develop resistance to the drugs, those new strains of resistant bacteria can in turn be transmitted to humans through contaminated meat, soil, and water [17]. Concerns over antimicrobial disease transmission have heightened as abattoirs and dairy operations are consolidated and more livestock are confined in closer quarters. The use of antibiotics as growth enhancers is particularly problematic because it entails applying low doses over long periods to large numbers of animals, potentially transforming the livestock into reservoirs for antibiotic resistant pathogens [18].

### Mass Production and *E. Coli *O157:H7

Competitive pressures of the global market also have incited the consolidation of food production. Along with the efficiencies of intensified production come increased opportunities for cross contamination – and significant challenges in tracing the original source of infections. Ground beef from a single cow may be mixed with that of hundreds of other cows at several different stops along the production process, from slaughter to processing to retail packaging. This consolidation is believed to have been a factor in the emergence and spread of diseases such as *Escherichia coli *O157:H7 [19].

In the winter of 1982, a series of outbreaks of enteric disease in Oregon and Michigan revealed the presence of *Escherichia coli *O157:H7, a serotype discovered in 1975 that had been identified in a human only once before [20,21]. Investigators tracked the source of the infection to undercooked beef patties served at fast food restaurants and ultimately, to a lot in Michigan that had supplied the outlets with ground beef. Over the next decade, several *E. coli *0157:H7 outbreaks, mostly tied to ground beef, cropped up across western and Midwestern U.S. In December 1992, more than 500 people in Washington, Idaho, California and Nevada developed the trademark symptoms, and four people died. The *E. coli *0157:H7 outbreak was associated once again with undercooked, contaminated beef patties served at a fast food restaurant. In response, the restaurant chain recalled more than a million beef patties and recovered about 20 percent, preventing an estimated 800 additional cases[22]. A team from the Centers for Disease Control identified one Canadian and five U.S. slaughter plants as potential sources of the contaminated lots, but they were not able to definitively pinpoint the source.

Today *E. coli *0157:H7 is a significant health concern in a growing number of regions around the world, particularly areas of Europe and North and South America; and South Africa and Japan. *E-coli *O157:H7 infections increasingly are linked with a range of meat and produce products, from salami to melons to lettuce. In most cases, produce is contaminated by water or soil containing feces, commonly from agricultural run-off. The bacteria can survive several months in standing water or frozen products. In February 2004, three cases of E. coli 0157:H7 infection in Okinawa, Japan were linked to hamburgers made from frozen ground beef purchased at a local U.S. military base. A subsequent investigation revealed the meat had been produced some six months earlier in the U.S. and also may have been responsible for several cases of infection that had been reported in California the previous August and September [23]. A 1991 outbreak traced to apple cider highlighted the microbe's ability to endure acidic conditions. Unwashed apples had been pressed in a mill, which then passed on the infection to subsequent batches. Investigators discovered the *E-coli *O157:H7 survived nearly three weeks in refrigerated, unpasteurized cider [24].

Conspiring to make *E. coli *O157:H7 an emerging threat in the international marketplace are its virulence and resilience, along with the relatively low doses required for infection, enabling ready transmission and enhancing opportunities for large outbreaks. Further contributing to the risk of spread is the growing list of contaminated produce. U.S.-grown radish sprout seeds, for example, were implicated in a massive outbreak in Japan. In the spring and summer of 1996, more than a dozen clusters of *E. coli *0157:H7 infection swept through central Japan, resulting in 10,000 cases – 6,000 among school children and the rest among factory workers [25].

### Uneven resources and Unknown Agents: *Cyclospora*

While global commerce offers the promise of boosting struggling economies by enabling them to participate in the global marketplace, realizing that potential is a complicated proposition. One critical issue is determining whether a nation's resources and land are best invested in crops that serve and rely on external markets. Another concern is whether the small growers that traditionally have been the backbone of the agricultural system in developing countries have the basic sanitary infrastructure necessary to develop products that can compete internationally. The situation becomes even more complex when it involves little-known, and consequently unpredictable, microbes such as *Cyclospora cayetanensis*.

When *C. cayetanensis *was first detected in humans in the late 1970s, it appeared to be confined mostly to tropical and sub-tropical areas of the developing world, affecting primarily children and people with compromised immune systems. On the occasions that cases of cyclosporiasis appeared in developed countries, it struck travelers who had visited areas where the disease was known to exist – and who presumably had been exposed to contaminated water. Humans are the only known host of the coccidian parasite, which is transmitted by oocysts excreted in feces that require at least several days outside the host to sporulate and become infective. It would become evident through a series of outbreaks in the 1990s that these properties enable *C. cayetanensis *to mature into an infectious agent while being transmitted on produce, apparently over long distances and several weeks.

In the spring of 1996, the CDC received reports of nearly 1,500 cyclosporiasis cases in the U.S. and Canada [26]. Investigators examined patterns among the outbreaks and determined that virtually all were linked to events at which fresh raspberries had been served. The raspberries, in turn, were traced to Guatemala. Generally, the symptoms of cyclosporiasis don't surface for at least a week; consequently by the time the initial outbreaks had been recognized, it was too late to establish the precise source of the contaminated berries. But the far-reaching nature of the outbreak suggested that a common practice among several suppliers, rather than a single farm, was responsible for the contamination [27]. Investigators concluded the most likely source of the infection was contaminated water used in some step of the berry-growing process.

In response to the findings, the Guatemalan Berry Commission implemented some measures targeting water and sanitation practices on the farms, and classified individual growers according to risk. But the efforts failed; the berry season in 1997 was a repeat of the year before – the CDC received reports of 41 clusters involving more than 1,000 cases from 13 U.S. states, the District of Columbia, and one Canadian province [27]. The investigation once again lead to Guatemala, which then suspended raspberry exports, incurring an estimated $10 million USD in lost income [28,29]. For the 1998 spring season, the U.S. FDA banned imports of fresh raspberries from Guatemala. That year, Canada, which had not banned the raspberries, once again experienced a series of outbreaks affecting more than 300 people in the Ontario area, but the U.S. did not, further establishing the link to Guatemala as the source of the infections [30].

Along with raspberries, *C. cayetanensis *infections have been associated with other produce, including basil, mesclun lettuce and snow peas [27,31]. In only a couple of cases – one involving basil, the other frozen raspberries – has the microbe been identified on the produce suspected of causing the outbreak. Similarly, the modes of contamination have been even more elusive. Cyclosporiasis' week-long incubation period, coupled with the fact that *C. cayetanensis *often travels on fresh produce that is long gone by the time the infection is discovered can make it difficult to identify the source and manner of the infections. Adding to the challenge is the fact that very low doses of exposure apparently are required for infection – consumption of just a couple raspberries can be sufficient[27].

### Product Innovations and the Global Palate: Listeria

Increasingly, appetites are bridging borders. Grocery stores feature "ethnic food" isles and delis are stocked with luxury imports from around the world, from foie gras to smoked duck. At the same time, food producers are developing "ready-to-eat" foods to meet consumers' demands for convenience. In 2002, processed goods made up nearly half of all agricultural exports [5]. Left behind in the whirl of innovation are food safety regulations drafted before many of these foods became popular, providing an opening for bacteria such as *Listeria monocytogenes*.

The *L. monocytogenes *bacterium, rare but also relatively dangerous, became a public heath concern in the '80s, when the illness was linked definitively with foods such as deli meats, smoked fish, fresh soft cheeses and pâté. At the same time, ready-to-eat foods, attractive for their convenience as well as their profit margins, were growing in popularity. But the additional steps entailed in processing present more opportunities for pathogens to be introduced into products. *L. monocytogenes *was first identified as a foodborne pathogen in 1953, when a woman who had consumed milk from an infected cow had stillborn twins. For the next few decades, however, it went largely unnoticed until several major outbreaks in the 1980s caught health officials' attention. In 2000, it was the pathogen most commonly associated with hospitalization in the U.S., and accounted for a third of reported pathogen-related deaths [32]. A study examining the 54 *L. monocytogenes *outbreaks reported around the world from 1970 to 2002 found that roughly one third occurred in the U.S. In more than 90 percent of the cases, contaminated meat or dairy products were identified as the source of the infections[32].

The challenges *L. monocytogenes *entails are particularly evident in the burgeoning world cheese market. In recent decades, the cheese varieties on offer have expanded from several dozen to several hundred, among them myriad soft cheeses and boutique artisanal cheeses – many of them potential vehicles for *L. monocytogenes*. The pathogen, which can survive refrigeration, can invade early on in the process and endure in raw cheeses, or reinfect a cheese after pasteurization. In Switzerland, between 1983 and 1987, 122 infections and 34 deaths were linked to Vacherin Mont D'Or cheese before officials discovered the microbe was lingering on the wooden shelves in the aging cellars, contaminating one batch after another [33]. In the Los Angeles area in 1985, 86 cases of *L. monocytogenes *infection linked to raw milk cheese resulted in 29 deaths, including 13 stillbirths and eight newborns [34]. In that instance, the infections were linked to a commercial cheese. But often queso fresco – a fresh, soft cheese made from unpasteurized or "raw" milk – is produced in private homes, making it difficult for health officials to enforce sanitary regulations.

Presently in the U.S., commercially manufactured cheeses must either be made from pasteurized milk or, if they are made from raw milk, be cured for a minimum of 60 days to outlast any remaining pathogens. However, the so-called "60-day" rule, developed in the 1950s before many of the cheeses it regulates existed, has come under scrutiny in recent years as it has become evident that a number of pathogens, including *Salmonella *and *E. coli *O157:H7, can withstand the 60-day aging period. Investigators demonstrated that *L. monocytogenes *can endure for up to 434 days[35]. Further complicating the issue is the fact that the pathogens can survive outside the food product on equipment or storage facilities and contaminate cheeses via that route, rendering the aging process moot. Discussions about modifying the 60-day rule are under way. Meanwhile, regulations require that raw milk cheeses must be labeled as such.

### International Safety Systems

Clearly domestic food safety, tracing and surveillance systems play a key role in stemming foodborne outbreaks. But the cross-border nature of commerce and thus infections also requires an effective international response. To that end, the World Health Organization has established the Global Public Health Intelligence Network (GPHIN), a web-based system that monitors news reports of infectious disease outbreaks around the world; Salm-Surv, a global network linking laboratories tracking the incidence of *Salmonella *and other foodborne diseases; the Global Outbreak Alert and Response Network (GOARN), which provides technical assistance within 24 hours to governments facing potential epidemics; and the International Food Safety Authorities Network (INFOSAN), which enables trans-border collaboration and assistance among food safety officials. While these networks are invaluable, ultimately their effectiveness relies substantially on individual nations' surveillance and diagnostic capabilities. Meanwhile, the WHO's International Health Regulations currently only require notification of outbreaks of cholera, yellow fever and plague. The 50-year-old regulations are being revised to cover all outbreaks of public health significance, including those with the potential to spread beyond borders, such as foodborne diseases. Until they are revised, the regulations provide little protection against the spread of such diseases.

The primary vehicles for addressing health matters as they relate to internationally traded goods are the World Trade Organization agreements. The Technical Barriers to Trade (TBT) and Sanitary and Phytosanitary (SPS) agreements address processes and standards for traded products. The SPS covers most potential vehicles for microbe "hitchhiking" – that is, products from farms and fields. Both agreements aim to provide some measure of predictability and reduce discrimination among trading countries by applying common standards to all trading partners. Under the SPS, the recognized sources for these standards are international organizations addressing food, plant and animal safety – respectively the Codex Alimentarius ("Food Law") Commission (run jointly by the World Health Organization and Food and Agriculture Organization of the UN), the International Plant Protection Commission (IPPC) and the Organisation for Animal Health (Office International des Épizooties, or OIE). While the standards are set forth as science based, some critics contend that the science is heavily influenced by the industry groups, such as those that attend Codex Alimentarius Commission meetings in large numbers [36]. Detractors maintain that the WTO and its consulting organizations are dominated by the major trading economies and generally serve the corporate interests of transnational companies rather than those of the public. While this can be debated, it is indisputable that the primary aims of the WTO agreements are to reduce – not erect – trade barriers.

## Conclusion

In response to the growing market pressures of global commerce, producers are scrambling to meet the challenge by making more diverse and better products. These constantly evolving dynamics of the global market are rendering existing safety systems outdated and in some cases simply impractical even as they are being adapted. The challenge of assuring the safety of the global food supply is a matter not only for public health but for private sector interests in the food industry. Nonetheless, the study and documentation of the complex changes taking place in food-related infections and the requisite health protections have fallen chiefly to the public sector. While the health and safety measures the World Health Organization and World Trade Organization provide are considerable, they are limited by the respective organizations' resources and priorities. Global agricultural products trade in 2002 was a $583 billion enterprise. The relevant industry science is geared toward discovery of new products or processing, quality assurance of specific products, and on occasion, creating an information base for use in standard setting for the industry. In view of conflict of interest, redirecting the scientific enterprise of industry towards investigating its own products seems an unlikely strategy. However, since the health and welfare of the consuming public is a common concern, additional investment of food industry proceeds in epidemiologic investigation, laboratory, and public health at local and global levels would seem a reasonable pathway. Networks such as INFOSAN will likely identify particular areas where arm's length investment by industry could help shore up public sector capacity in resource poor economies.

## Competing Interests

The author(s) declare that they have no competing interests.

## Authors' contributions

JH reviewed the literature and drafted the manuscript. AMK conceived of this review, surveyed the literature and helped to draft and edit the manuscript. Both authors read and approved the final manuscript.
